# The Spectral Compositions of Light Changes Physiological Response of Chinese Cabbage to Elevated Ozone Concentration

**DOI:** 10.3390/ijms23062941

**Published:** 2022-03-09

**Authors:** Andrzej Skoczowski, Jakub Oliwa, Iwona Stawoska, Magdalena Rys, Maciej Kocurek, Ilona Czyczyło-Mysza

**Affiliations:** 1 Institute of Biology, Pedagogical University of Krakow, Podchorążych 2, 30-084 Kraków, Poland; andrzej.skoczowski@up.krakow.pl (A.S.); iwona.stawoska@up.krakow.pl (I.S.); 2 The Franciszek Górski Institute of Plant Physiology, Polish Academy of Sciences, Niezapominajek 21, 30-239 Kraków, Poland; m.rys@ifr-pan.edu.pl (M.R.); i.czyczylo@ifr-pan.edu.pl (I.C.-M.); 3 Institute of Biology, The Jan Kochanowski University, Uniwersytecka 7, 25-406 Kielce, Poland; maciej.kocurek@ujk.edu.pl

**Keywords:** *Brassica rapa* subsp. pekinensis, photosynthesis, chlorophyll *a* fluorescence, Raman spectroscopy, LED light

## Abstract

The effects of ozone combined with other environmental factors remain an important topic of the research, both in connection with climate change and the possibility of using modern solutions in horticulture. In our experiment, we compared the influence of ozone (100 ppb) on photosynthesis and changes in the pigment composition of Chinese cabbage (*Brassica rapa* subsp. pekinensis) leaves depending on the spectral composition of light. We used white LED light (WL), a combination of red + green + blue (RGBL) with a dominant red component and white +blue (WBL) with a dominant blue component in comparison with the classic sodium lamp lighting (yellow light—YL). The values of the parameters describing the light-dependent phase of photosynthesis and the parameters of the gas exchange, as well as non-photosynthesis pigment contents, show that the spectral composition strongly differentiates the response of Chinese cabbage leaves to ozone. In general, the efficiency of photochemical reactions was the highest in YL, but after O_3_ fumigation, it decreased. In plants growing in WL and WBL, the increase of O_3_ concentration stimulated light photosynthesis reactions and led to the enhancement of transpiration, stomatal conductance and intracellular CO_2_ concentration. Changes in photosynthetic activity were accompanied by an increase in the content of anthocyanins and flavonols.

## 1. Introduction

Abiotic environmental factors play a key role in the growth and development of all plants and, in the case of cultivated species, also have an impact on their value in use. Plant metabolic activity—in particular, the photosynthesis and the synthesis of secondary metabolites—are strongly dependent on light conditions. Both the intensity and spectral composition of sunlight undergoes constant fluctuations, which are associated with dynamic changes in natural atmospheric components (CO_2_, water vapour and suspended dust), as well as an increase in air pollution, e.g., by ozone. In the case of vegetable growing, in addition to photon flux density, the spectral composition of light is also important, as it can be modified and adapted to optimise plant growth to obtain higher yields in shorter time. This modification of the light spectrum can significantly and favourably affect photochemical reactions of photosynthesis, as well as the anatomical and morphological structures of cultivated plants [1]. Long-term growth in low-light conditions (shadow) leads to a decrease in the plant’s energy resources, which results in a reduction in photosynthetic activity and a decrease in biomass. For this reason, the shade avoidance response is often found in many plant species. It manifests, among others, in the elongation of stems and petioles (hyponastic response), thanks to which, the plant can compete better for light. It has long been known that this mechanism is regulated by changing the red/far-red (R/FR) value ratio in the light spectrum. However, also, blue light (and, therefore, the blue/red ratio value) can significantly affect the shadow avoidance response [2].

Ozone is usually treated as a pollutant, which, in high concentrations, adversely affects the development of plants, contributing to the reduction of the primary productivity of both natural ecosystems and crops [3,4,5]. Many crop species (e.g., tobacco, string beans, soybeans, wheat and cowpeas) are sensitive to high ozone concentrations [6,7,8]. The negative effect of an elevated O_3_ concentration on crop plants, which is manifested by stomatal limitation, increased reactive oxygen species (ROS) synthesis and, consequently, inhibition of photosynthesis, has been repeatedly confirmed [6,9,10]. Previous studies presented a diverse experimental approach and were carried out under controlled (climatic chambers), semi-controlled (greenhouses, top-openchambers) and outdoor conditions [9]. However, only a small part of the experiments considered the synergistic effects of ozone and other abiotic factors, especially light [11,12,13].

The tolerance of elevated ozone concentrations in individual plant species differs fundamentally. It is associated with the ability to regulate the stomatal conductance and change in oxidative signalling, as well as activation of defence mechanisms [3,4,5,14]. As a result, acclimatisation to a new O_3_ concentration and an increase in photosynthesis efficiency were observed in some species (e.g., apple trees) [15]. In turn, in *Brassica oleracea* L., ozone fumigation caused growth acceleration and permanent changes in the antioxidant system [14]. The use of short exposure to O_3_ may increase plant tolerance to photoinhibition [12]. Antioxidants such as anthocyanins and flavonoids play an important role in this process, as they protect the photosynthetic apparatus from excess light energy [16,17].

The measurement of Chl*a* fluorescence kinetics is a non-destructive method often used to analyse PSII responses to environmental stress [18,19,20,21]. It ensures obtaining, in a short time, parameters describing the efficiency of absorption, distribution and dissipation of light energy within PSII that reacts quickly with changes in abiotic factors, e.g., an increase in ozone concentration [15,22,23,24]. It has long been successfully used for the early detection of changes in the light phase of photosynthesis under the influence of O_3_ before visible signs of stress appear [24]. The analysis of Chl*a* fluorescence kinetics was also used to assess the interaction of ozone and other environmental stresses, e.g., high light [12]. This method is also particularly useful for assessing changes occurring in PSII under the influence of the different spectral compositions of light [25,26]. A complementary method that allows determining the effect of O_3_ on photosynthesis is gas exchange analysis, i.e., net photosynthesis, as well as transpiration, stomatal conductance and intracellular CO_2_ concentration [6,13,24]. In turn, FT-Raman spectroscopy and light reflectance analysis allow to assess the chemical compositions of plants—in particular, the estimation of the total protective pigments content (such as anthocyanins, flavonols and carotenoids) without affecting the tissue structure [27,28,29,30,31].

The goal of this experiment was to investigate how the spectral composition of light influences the photosynthetic response and photosynthetic pigment contents of Chinese cabbage leaves exposed to an elevated O_3_ concentration (100 ppb). As a light source, we used LED matrices emitting white (WL), white–blue with a dominant blue component (WBL) and red–green–blue with a dominant red component (RGBL), respectively, and sodium lamp emitting yellow light (YL).

## 2. Results

### 2.1. Morphological Changes

**Figure 1:**
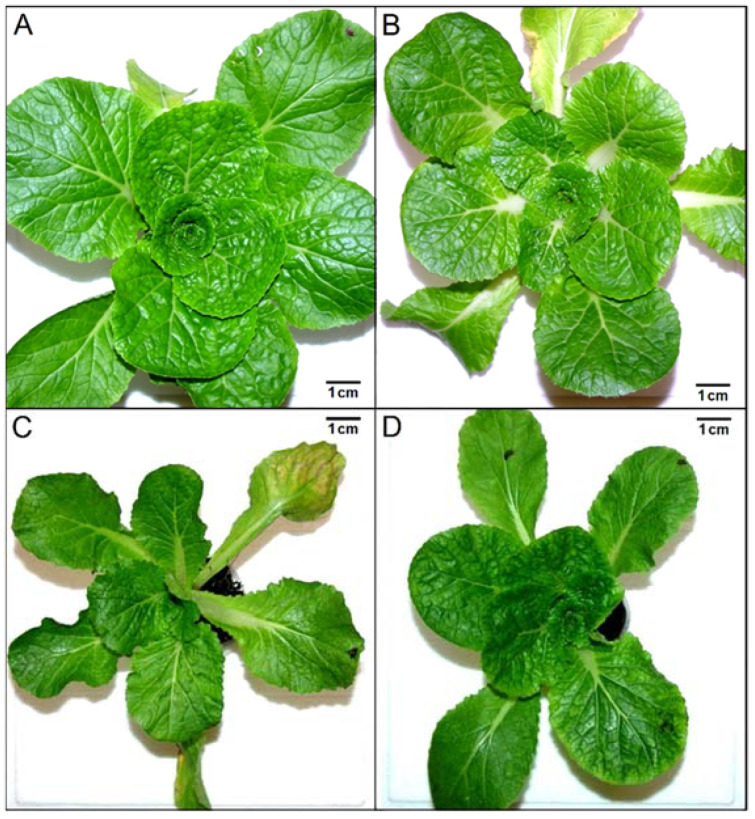
Typical plants of *Brassica rapa* subsp. pekinensis after 21 days of growth in 100-ppb O_3_ in different light spectra: (**A**) yellow light (sodium lamp, YL), (**B**) white light (WL), (**C**) light with a dominant blue component (WBL) and (**D**) light with a dominant red component (RGBL).

Plants after 21 days of exposure to 100-ppb O_3_ depending on the spectral composition of light are shown in Figure 1. After O_3_ exposure, no symptoms typical of ozone stress, i.e., chlorosis or necrosis, were observed. Ozone plants growing in YL and WL, Figure 1A and Figure 1B, respectively, had a much larger leaf blade size than in RGBL and WBL. In addition, WBL plants showed an increase in the length of petioles (Figure 1C).

### 2.2. Chlorophyll a Fluorescence

The maximum photochemical yield of PSII, measured as Fv/Fm in all ozonated and control plants, varied slightly (Table 1). Despite slight differences in the Fv/Fm values, WL, RGBL and WBL plants showed a statistically significant increase in this parameter after 21 days of O_3_ fumigation. The performance index of PSII (PI_ABS_) best differentiated the response of plants to the spectral composition of light and exposure to ozone (Table 1). In YL plants under the influence of O_3_, there was a decrease in PI_ABS_ value, while, in plants at other light spectra, a significant increase in this parameter was observed (especially in RGBL).

Phenomenological energy fluxes per excited cross-section such as RC/CSm and ABS/CSm were generally not significantly different after treatment with O_3_ in plants growing in WL, RGBL and WBL (exception RC/CSm in RGBL) but fell in YL (Table 1). Additionally, TR_0_/CSm and ET_0_/CSm values describing trapping and energy transfer in PSII were reduced in YL after fumigation, and in other spectral compositions, they increased or remained unchanged. Energy dissipation by PSII estimated based on DI_0_/CSm decreased in ozonated plants growing in WL, RGBL and WBL compared to controls but did not change in YL.

### 2.3. Gas Exchange Measurements

Net photosynthesis (*P*_N_) slightly decreased after O_3_ fumigation in YL and WBL plants, while WL plants showed a P_N_ increase. There were no significant differences in plants growing in RGBL. Generally, the *P*_N_ value of control plants in WL was the lowest and, in YL even after fumigation, remained the highest of all treatments. Values of the parameters connected with gas exchange, i.e., transpiration (*E*), stomatal conductance to water (*g*_s_) and intracellular CO_2_ concentration (*C*_i_) decreased under the influence of O_3_ in YL and increased significantly in the other spectral compositions of light (Table 2). After 21 days of exposure to O_3_, the highest values of most of the above parameters were recorded in plants growing in RGBL.

### 2.4. Reflectance Measurements

**Figure 2:**
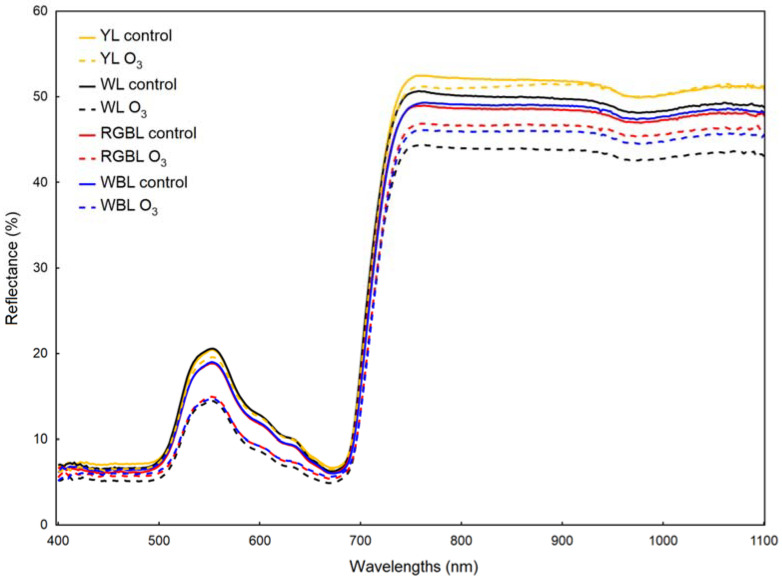
Leaf reflectance spectra of *Brassica rapa* subsp. pekinensis after 21 days of growth in 100-ppb O_3_ or not fumigated (control) in different light spectra: YL—yellow light (sodium lamp), WL—white light, RGBL—light with a dominant red component and WBL—light with adominant blue component. Average values from 7 independent replications.

The spectrum of reflectance from leaves in the visible region (peak around 550 nm) was reduced after O_3_ fumigation in plants growing in WL, RGBL and WBL (Figure 2). The percentage reflectance values in the infrared region of spectrum (NIR) were variable, and the largest decrease under ozone in the WL plants spectrum occurred. In the whole 400–1100-nm range analysed, the spectra of YL plants did not change significantly after O_3_ exposure (Figure 2).

The spectral composition of light significantly affected the changes in the content of protective dyes such as anthocyanins and flavonols. The total anthocyanins pool expressed by the ARI_2_ value increased under the influence of O_3_ in plants from all spectral compositions, except for RGBL, with the highest increase observed in YL plants (Table 3). After 21 days of O_3_ treatment, the anthocyanin content in YL and WL plants was similar. A very large increase in the content of flavonols (FRI) after ozone exposure took place in leaves growing under WBL. In the case of YL, WL and RGBL plants, this increase was significantly smaller (Table 3). The structure independent pigment index (SIPI) did not differ between treatments. Additionally, tissue hydration (water band index—WBI) was not dependent on light conditions and exposure to ozone (Table 3).

### 2.5. Chlorophyll Content

The chlorophyll content expressed in SPAD units was similar for all O_3_-treated and control plants, regardless of the light spectrum (Table 4). Only on the bottom side of the leaves in YL, a slight decrease in the content of Chl after ozonation was observed.

### 2.6. FT-Raman Spectroscopy Measurements

**Figure 3:**
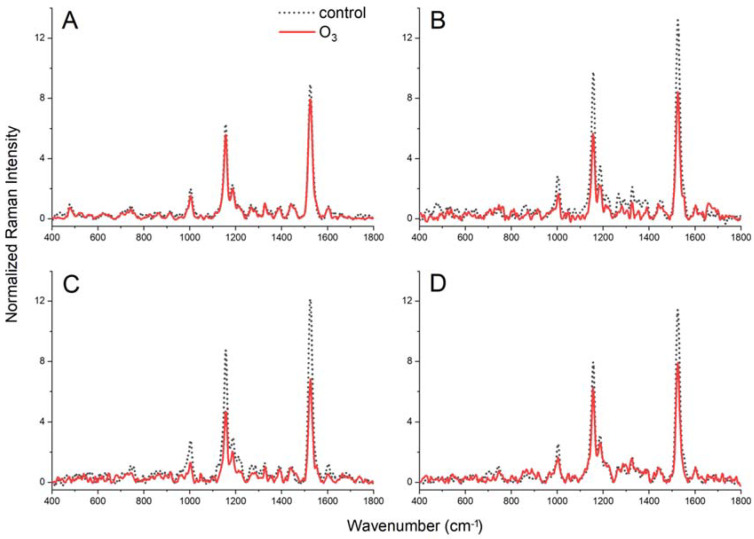
FT-Raman spectra, normalised according to 1441 cm^−1^, obtained for lyophilised leaves of *Brassica rapa* subsp. pekinensis growing in different light spectra: (**A**) yellow light (sodium lamp, YL), (**B**) white light (WL), (**C**) light with a dominant blue component (WBL) and (**D**) light with a dominant red component (RGBL). Solid line—spectra obtained after 21 days of ozone fumigation and dashed line—spectra for non-fumigated plants (control). Particular spectra represent the average values of 10 replications.

Table S1 presents the identification of chemical compounds assigned to bands in the obtained Raman spectra shown in Figure 3. The most characteristic are bands derived from carotenoids localised at 1005, 1158 and 1525 cm^−1^, the so-called carotenoid triplets [32,33]. The peaks observed at 1158 and 1525 cm^−1^ are attributed to –C–C and C=C-stretching vibrations of the polyene chain, respectively. Furthermore, the position of the band at 1525 cm^−1^ suggests that the carotenoid moiety possesses nine conjugated C=C bonds. It should also be noted that the band at 1158 cm^−1^ is formed as an imposition, with the weaker band deriving from deformation vibrations δ(CH) in phenolic compounds [34]. The low-intensity band at 1005 cm^−1^ reflected –CH_3_ groups attached to the main chain coupled with –C–C bonds [32,35].

In non-fumigated YL plants, the intensity of the carotenoid triplets was weaker compared to WL, WBL and RGBL plants. After O_3_ treatment, the decrease of the carotenoid triplet intensity was observed in WL, WBL and RGBL (up to 50% for WL and WBL compared to the control), and a significantly lower decrease was registered in YL (app. 12%). Furthermore, at frequencies 746, 1186, 1282 and 1552 cm^−1^, vibrations characteristic of chlorophylls were identified [32,34,35,36,37]. However, it is important to add that the band localised at 1552 cm^−1^ originates from the superposition of chlorophylls and phenolic compounds [38]. In the range of 1250–1400 cm^−1^, there are several peaks that indicate the presence of stretching and deformation vibrations specific to the -CH, -CH_2_ and -CH_3_ groups that are responsible for building chains of fatty compounds [37,39,40]. In the presented spectra, the bands at 1602 cm^−1^ originating from phenyl ring vibrations are also visible, which endorses the phenolic compound presence in examined plant samples.

**Figure 4:**
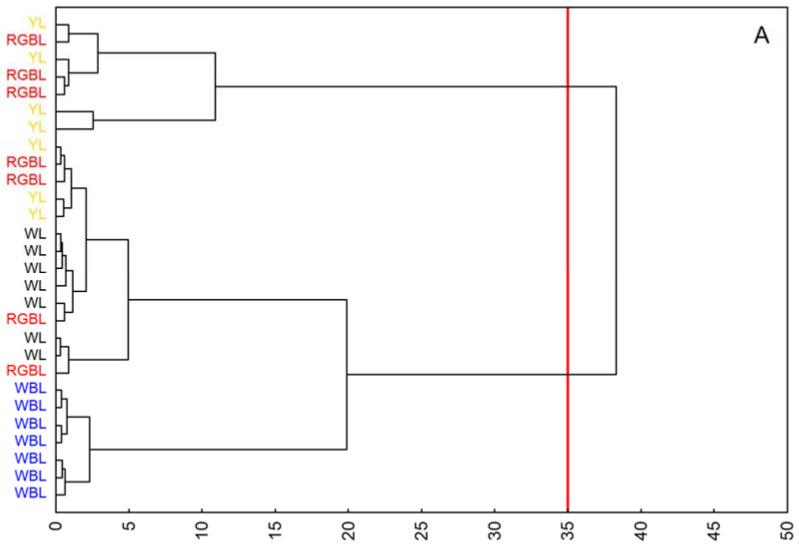

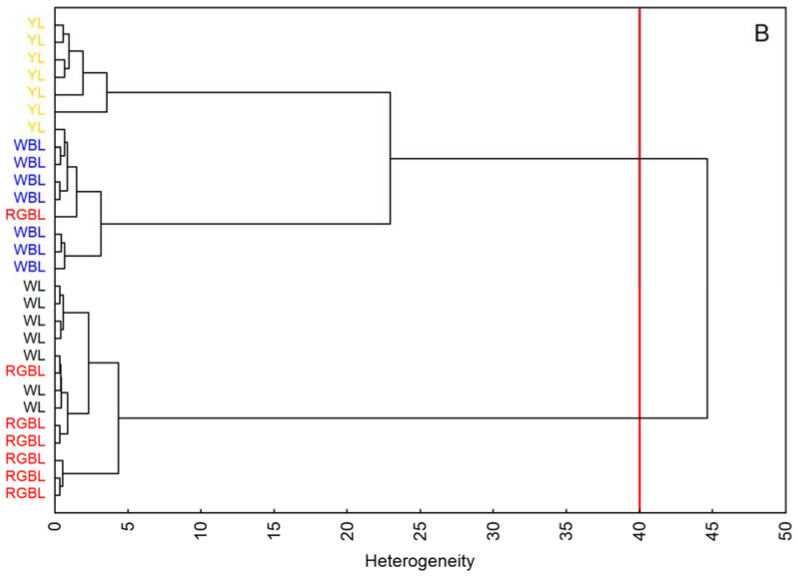
Dendrograms of a hierarchical cluster analysis according to Ward’s algorithm of the FT-Raman spectra obtained for the leaves of *Brassica rapa* subsp. pekinensis growing under different spectral compositions of light. (**A**) Control plants and (**B**) plants fumigated by ozone (21 days, 100 ppb). The red line indicates the number of statistically significant groups to which individual objects have been classified.

The effect of the spectral composition of light and ozone on the chemical composition of the leaves was investigated using a hierarchical similarity analysis (Figure 4A and Figure 4B, respectively). Increased ozone concentrations in different spectral compositions of light differentiate the chemical compositions of leaves more than the light colour itself. In the case of ozone-treated plants, two main clusters that connect leaves of similar chemical composition could be distinguished: the first for YL and WBL and the second for WL and RGBL (Figure 4B).

## 3. Discussion

### 3.1. Morphological Differences

A change in the spectral composition of light significantly affects a plant’s anatomy and morphology [41]. In our experiment, dominance of the selected wavelength in the spectrum (blue—WBL or red—RGBL) in combination with an increased concentration of O_3_ resulted in differences in plant habits (size reduction of the leaf blade) compared with plants from YL and WL (Figure 1). Moreover, WBL plants showed petiole elongation, which should be explained by a hyponastic response [2]. A similar effect on leaf development was demonstrated in *Brassica oleracea* with an increase in the value of the R/FR ratio in the light spectrum [42]. Sometimes, an increased ozone concentration may have a positive effect on the rosette size and leaf number in *Brassica* plants [14]. However, the simultaneous effects of ozone and qualitative changes in lighting can also induce stress reactions in plants [12].

### 3.2. The Physiological Effect of Ozone and Yellow LIGHT (YL) on Plants

In plants growing in YL, ozone induced an increased production of compounds aimed at protecting PSII against the adverse effects of ROS—increase in ARI_2_ and FRI values (Table 3). Despite the synthesis of antioxidants in YL plants, ozone reduced the efficiency of the photosynthesis light phase, which was manifested in a decrease in the density of active PSII reaction centres calculated on the surface of the excited sample (RC/CSm), a decreased energy absorption by the excited photosynthetic surface (ABS/CSm). This was confirmed by the parameters of the trapping efficiency and energy transport by PSII of the excited sample (TR_0_/CSm and ET_0_/CSm). The PI_ABS_ value also decreased. The changes in PI_ABS_ perfectly reflect the overall functioning of PSII, because they combine information about the number of active RCs per Chl molecule and the initial reactions of the light phase with data on the electron flux through the RC [18]. For this reason, this parameter allows a comprehensive assessment of the state of PSII and is interpreted as a measure of the internal strength of the plant that allows it to counteract the stress symptoms [43].

Sunlight can change the reaction of leaves to O_3_ mainly by reducing the rate of electron transport in PSII [12]. In our research, exposure to an elevated O_3_ concentration has slightly reduced the efficiency of photosynthesis in *B. rapa* leaves growing in YL (Table 2), which is often the observed effect of ozone. We suggest that the decrease in net photosynthesis in this case was due to stomatal limitation, which was aimed at reducing the intensity of O_3_ diffusion [44,45]. This corresponds to a decrease in stomatal conductance, intercellular CO_2_ concentration and transpiration (Table 2). Ozone diffusion by stomata and the local accumulation of excess ROS is also evidenced by a slight decrease in the chlorophyll content observed only on the bottom side of the leaves growing in YL (Table 4). Despite this, no negative changes in the leaf morphology and plant habit were observed.

### 3.3. Physiological Response of Plants to Ozone under LED Lighting

In contrast to YL plants, we observed a positive effect of ozone in Chinese cabbage leaves in WL, RGBL and WBL on the photosynthesis parameters. After O_3_ fumigation, PI_ABS_ values in RGBL increased almost twice, which shows a significant increase in energy conversion efficiency in PSII under the influence of O_3_ [46]. However, a large increase in the value of PI_ABS_ and a slight increase in the value of Fv/Fm were visible in all plants growing under the LED light, despite the lack of differences in chlorophyll content (Table 4) and the density of PSII reaction centres on chlorophyll (RC/CSm—Table 1). This was accompanied by an increase in the efficiency of energy transfer by excited chlorophyll (ET_0_/CSm) and a decrease in energy dissipation by chlorophyll (Di_0_/CSm). Thus, in white light and in the spectrum with predominantly red or blue colour, ozone not only did not interfere with the photosynthesis light reaction processes but even increased the efficiency of the light-dependent reactions. Combined with the increase in ARI_2_ values in WL and WBL plants and FRI in RGBL and WBL plants (Table 3), this indicates the involvement of de novo synthesised pigments (anthocyanins and flavonols) in better energy distribution in PSII after exposure to ozone. The accumulated pigments can act as an antioxidant system that prevents the generation of ROS and as a photoprotective compound [47,48]. Ozone, absorbed by stomata, quickly penetrates the intercellular spaces and interacts with water and other compounds in the apoplast (e.g., thiols or phenols), inducing the formation of reactive oxygen species (ROS), the excess of which leads to membrane lipid ozonolysis and protein damage [49,50]. This stimulates the increased synthesis of compounds whose function is to protect thylakoid chloroplast membranes against damage by ROS [47,51,52]. This is particularly important when exposing P680 triplets to singlet oxygen [53,54]. The rapid synthesis of anthocyanins and flavonols supports the inhibition of ROS production and stabilises chloroplast membranes [55]. Interestingly, at the same time, it was noticed that O_3_ fumigation led to a decrease in the intensity of the carotenoid triplets compared to the control (Figure 3). This indicates a reduction in the carotenoid pool and suggests their minor role in the response to ozone stress in WL, RGBL and WBL plants.

Low values of *E*, *g*_s_ and *C*_i_ in control plants growing in WL, RGBL and WBL increased significantly after 21 days of exposure to O_3_ (Table 2). In WL plants, which, under control conditions, achieved significantly lower *P*_N_ values than YL and RGBL, this value increased after fumigation. This indicates an increase in the activity of photosynthesis, probably due to an increase in the supply of energy from light reactions (Table 1). Additionally, according to Kleiber et al. [13], lettuce, whose seeds were ozonated, showed the largest increase in photosynthetic activity in WBL. In addition, the positive effect of ozone was dependent on the light spectral composition, and the highest effect was observed for WL.

## 4. Materials and Methods

### 4.1. Plant Materials

Sprouted Chinese cabbage seeds (*Brassica rapa* subsp. pekinensis) variety Optico F1 were sown into seedbeds with soil substrate and placed in a phytotron chamber for a period of three weeks. Seedlings grew under sodium lamps widely used in greenhouse and tunnel crops. The light source was a Philips SON–T AGRO 400W lamp (Agro Care, Rilland, The Netherlands), and the photon flux density was 300-µmol quantum m^−2^ s^−1^. The growth temperature was set at 18 °C/14 °C and photoperiod at 12h/12h (day/night, respectively). The relative humidity (RH) was kept at 80%. When the seedlings reached stage 4/5 of the leaves, the plants were divided into four groups: one part was left under yellow light (YL—sodium lamp), and the other was transferred to white light (WL), white light supplemented with blue light (WBL) and red-green-blue light (RGBL). The light sources were LED matrices made using white, blue and RGB LEDs, 1W each. All matrices consisted of 225 diodes. In the WL spectrum, the intensity ratio of blue/red light ≅ 1 and in WBL > 1, while in RGBL < 1. The spectra of the light sources were determined by a Mini-spectrometer TM-VIS/NIR C10083CAH (Hamamatsu, Japan) and are shown in Figure S1. All other parameters such as temperature, day length, RH and PFD remained unchanged. Plants were fumigated with 100-ppb ozone for three weeks, 8 h per day (during the light period). The Aqua Medic Ozone 50 generator (Aqua Medic, Poznan, Poland) was used to generate ozone, and the O_3_ level was controlled using a 49C Photometric Ozone Analyser (Thermo Environmental Instruments, Franklin, MA, USA). Control plants (not treated with ozone) grew under analogous lighting, temperature and humidity conditions as the plants treated with ozone. Experiments were carried out in 7 replications (1 single plant was the replication). All measurements were performed after 21 days of plant growth in 100-ppb O_3_ or under the control conditions for each type of lighting.

### 4.2. Chlorophyll a Fluorescence

Measurements of the Chl*a* fluorescence kinetics parameters were made with a Handy-PEA fluorometer (Hansatech Instruments, Narborough, UK), according to Strasser et al. [56]. Each time, the leaf blade fragments were acclimated to darkness for 20 min. The Chl*a* fluorescence was induced with 3500-μmol quantum m^−2^ s^−1^ radiation (650-nm peak wavelength, 22-nm half-width spectral line). The data were read in the PEA Plus program (Hansatech Instruments, Narborough, UK). Fluorescence parameters were calculated automatically: maximum quantum yield of PSII (Fv/Fm) and performance index of PSII based to absorption (PI_ABS_). Moreover, the following phenomenological indices of energy flow converted to the excited surface of the leaf were determined: density of reaction centres (RC/CSm), absorption of light energy (ABS/CSm), excitation energy trapped in PSII reaction centres (TR_0_/CSm), energy used for electron transport (ET_0_/CSm) and energy dissipated from PSII (DI_0_/CSm).

### 4.3. Gas Exchange Measurements

Photosynthesis rate (*P*_N_), stomatal conductance (*g*_s_), transpiration rate (*E*) and internal CO_2_ concentration (*C*_i_) values were measured using an LI-COR 6400 (Li-Cor, Lincoln, NE, USA) infrared gas analyser in an open system. All measurements were made on the seventh leaf.

### 4.4. Chlorophyll Content

Measurements of the Chl content were taken using the SPAD 502 Chlorophyll Meter (Konica Minolta, Tokyo, Japan). The SPAD values were calculated based on the amount of radiation transmitted by the leaf at 650 and 940 nm. For each plant, the 7th leaf was selected, on which the Chl content was measured 3 times, and then, the internal mean was calculated.

### 4.5. Reflectance Measurements

The leaf reflectance was measured using the LI-1800 spectroradiometer (Li-Cor, Lincoln, NE, USA) with a−12 S external integrating sphere in the range 400–1100 nm [57]. Based on the spectra obtained, the following reflection parameters were calculated:

- Anthocyanin Reflectance Index (ARI_2_) = [(R_550_^−1^) − (R_700_^−1^)] R800, Gitelson et al. [58];
- Flavonol Reflectance Index (FRI) = [(R_410_)^−1^ − (R_460_)^−1^] R800, Merzlyak et al. [59];
- Structure-Insensitive Pigment Index (SIPI) = (R_800_ − R_445_) (R_800_ + R_680_) − 1, Peñuelas et al. [60];
- Water Band Index (WBI) = R_900_∙(R_970_)^−1^, Peñuelas et al. [61];
  where R_x_—means the intensity of reflectance at a specific wavelength x.

### 4.6. FT-Raman Spectroscopy Measurements

FT-Raman measurements were performed on lyophilised leaves of Chinese cabbage using a Nicolet NXR 9650 Fourier-Transform Raman spectrometer (Thermo Scientific, Waltham, MA, USA) equipped with a Nd:YAG3+ laser emitting a beam at the 1064-nm wavelength and an InGaAs detector. The measurements were done at room temperature, an aperture of 80 and a spectral resolution of 4 cm^−1^. All spectra were recorded with a laser power of 0.4 W and analysed in the range of 400–2000 cm^−1^. The number of accumulations for each spectrum was equal to 128 scans. Each measurement was done in 10 repeats and averaged. The spectra were baseline-corrected and normalised to the 1441-cm^−1^ band, whichis typical for C-H vibrations originating most likely from the CH_3_, CH_2_, and CH functional groups in lipids, amino acid side chains of the proteins and carbohydrates [62,63]. The analysis of the spectra was carried out using Omnic 8 (Thermo Scientific, Waltham, MA, USA) and OriginPro 2017 (OriginLab Corporation, Northampton, MA, USA) software packages for Windows.

Similarities between FT-Raman spectra were studied using hierarchical cluster analysis using the Statistica software package 13 (TIBCO Software, Palo Alto, CA, USA). The cluster analysis was performed for the whole wavenumber range using Ward’s algorithm. The spectral distances were calculated with the standard algorithm.

### 4.7. Statistical Analysis

The Chl*a* fluorescence, gas exchange and leaf reflectance parameters were analysed using the program Statistica 13 (TIBCO Software, Palo Alto, CA, USA) using a one-way or multifactorial analysis of variance (ANOVA). The significance of the differences between averages was tested using Duncan’s test or Tukey’s test (for various N) at a significance level of *p* ≤ 0.05.

## 5. Conclusions

The photosynthetic activity of plants exposed to an elevated ozone concentration depends on light conditions. This translates into the processes of growth and development and, in the cultivated species, also, their value in use.

White light (WL) and spectrum with a predominant red (RGBL) or blue colour (WBL) increases plants’ ozone tolerance. Especially in plants growing in WL and WBL, exposure to an elevated O_3_ concentration stimulates photosynthesis light reactions and leads to an increase in transpiration, stomatal conductance and intracellular CO_2_ concentration, which, however, does not correspond directly with the morphological features of plants.

The spectral composition strongly differentiates the plant response to ozone; therefore, when conducting research on the influence of this factor on the physiological processes of plants in controlled conditions, it is necessary to precisely specify the quality parameters of the lighting used.

## Figures and Tables

**Table 1:** Chlorophyll *a* fluorescence kinetics parameters of *Brassica rapa* subsp. pekinensis growing in a different light spectrum: YL—yellow light (sodium lamp), WL—white light (LED), RGBL—red + green + blue light (LED), WBL—white + blue light (LED), treated with ozone (100 ppb, 21 days) or not fumigated (control). Average values (±SD) marked with the same letters in a row do not differ significantly according to Duncan’s test, *p* ≤ 0,05, *n* = 7.

Chlorophyll *a* Fluorescence Parameter	Treatment							
	YL		WL		RGBL		WBL	
	Control	O_3_	Control	O_3_	Control	O_3_	Control	O_3_
Fv/Fm	0.845 ^a^ ± 0.014	0.826 ^ab^ ± 0.012	0.823 ^bc^ ± 0.009	0.841 ^a^ ± 0.014	0.819 ^c^ ± 0.013	0.840 ^a^ ± 0.009	0.803 ^d^ ± 0.009	0.817 ^c^ ± 0.011
PI_ABS_	2.600 ^bc^ ± 0.259	2.130 ^de^ ± 0.208	1.781 ^ef^ ± 0.212	2.773 ^b^ ± 0.232	1.803 ^ef^ ± 0.190	3.395 ^a^ ± 0.266	1.575 ^f^ ± 0.214	2.387 ^cd^ ± 0.228
RC/CSm	1271 ^a^ ± 46	1095 ^c^ ± 41	1056 ^cd^ ± 33	1103 ^bc^ ± 42	1027 ^de^ ± 40	1166 ^b^ ± 41	992 ^de^ ± 44	964 ^e^ ± 38
ABS/CSm	3359 ^a^ ± 84	2988 ^d^ ± 74	3092 ^bc^ ± 85	3114 ^b^ ± 90	2868 ^de^ ± 76	2966 ^d^ ± 80	2813 ^ef^ ± 79	2730 ^f^ ± 72
TR_0_/CSm	2837 ^a^ ± 74	2492 ^c^ ± 78	2543 ^bc^ ± 82	2619 ^b^ ± 81	2348 ^d^ ± 84	2491 ^c^ ± 79	2261 ^d^ ± 76	2239 ^d^ ± 69
ET_0_/CSm	1561 ^a^ ± 62	1326 ^b^ ± 70	1324 ^b^ ± 59	1553 ^a^ ± 66	1223 ^bc^ ± 70	1541 ^a^ ± 64	1146 ^c^ ± 68	1292 ^b^ ± 73
DI_0_/CSm	521 ^bc^ ± 18	495 ^cd^ ± 20	549 ^ab^ ± 21	494 ^cd^ ± 18	519 ^c^ ± 20	474 ^d^ ± 18	551 ^a^ ± 14	490 ^cd^ ± 19

**Table 2:** Gas exchange parameters of *Brassica rapa* subsp. pekinensis growing in a different light spectrum and treated with ozone (100 ppb, 21 days) or non-fumigated (control). YL—yellow light (sodium lamp), WL—white light (LED), RGBL—red + green + blue light (LED) and WBL—white + blue light. Average values (±SD) marked with the same letters in the row do not differ significantly according to Duncan’s test, *p* ≤ 0.05, *n* = 7.

Parameter	Treatment							
	YL		WL		RGBL		WBL	
	Control	O_3_	Control	O_3_	Control	O_3_	Control	O_3_
*P*_N_ (µmol CO_2_ m^−2^ s^−1^)	8.73 ^a^ ± 0.80	7.57 ^b^ ± 0.82	2.99 ^e^ ± 0.77	5.20 ^cd^ ± 0.89	7.64 ^b^ ± 0.94	6.25 ^bc^ ± 1.73	4.57 ^c^ ± 0.69	3.77 ^de^ ± 0.85
*E* (mol H_2_O m^−2^ s^−1^)	0.97 ^c^ ± 0.24	0.56 ^d^ ± 0.22	0.52 ^d^ ± 0.19	1.81 ^b^ ± 0.49	2.20 ^b^ ± 0.43	3.24 ^a^ ± 0.28	0.70 ^cd^ ± 0.27	2.34 ^b^ ± 0.56
*g*_s_ (mmol H_2_O m^−2^ s^−1^)	0.12 ^bc^ ± 0.02	0.07 ^d^ ± 0.01	0.02 ^e^ ± 0.00	0.09 ^cd^ ± 0.02	0.10 ^bc^ ± 0.02	0.20 ^a^ ± 0.03	0.03 ^e^ ± 0.01	0.15 ^ab^ ± 0.03
*C*_i_ (µmol CO_2_ mol)	254 ^c^ ± 24	171 ^d^ ± 23	159 ^d^ ± 26	282 ^bc^ ± 23	249 ^c^ ± 20	309 ^ab^ ± 26	146 ^d^ ± 23	333 ^a^ ± 24

**Table 3:** Reflectance parameters from the leaves of *Brassica rapa* subsp. pekinensis growing in a different light spectrum and treated with ozone (100 ppb, 21 days) or non-fumigated (control). YL—yellow light (sodium lamp), WL—white light (LED), RGBL—red + green + blue light (LED) and WBL—white + blue light. Average values (±SD) marked with the same letters in the row do not differ significantly according to Duncan’s test, *p* ≤ 0.05, *n* = 7.

Reflectance Parameter	Treatment							
	YL		WL		RGBL		WBL	
	Control	O_3_	Control	O_3_	Control	O_3_	Control	O_3_
ARI_2_	0.555 ^d^ ± 0.021	0.728 ^a^ ± 0.002	0.686 ^b^ ± 0.001	0.748 ^a^ ± 0.001	0.628 ^c^ ± 0.003	0.595 ^cd^ ± 0.002	0.486 ^e^ ± 0.005	0.588 ^d^ ± 0.004
SIPI	0.763 ^a^ ± 0.012	0.771 ^a^ ± 0.002	0.767 ^a^ ± 0.001	0.788 ^a^ ± 0.022	0.774 ^a^ ± 0.017	0.781 ^a^ ± 0.004	0.772 ^a^ ± 0.024	0.770 ^a^ ± 0.006
FRI	0.799 ^d^ ± 0.011	1.051 ^b^ ± 0.002	0.393 ^g^ ± 0.005	0.493 ^f^ ± 0.003	0.657 ^e^ ± 0.003	0.957 ^c^ ± 0.011	0.725 ^de^ ± 0.022	1.815 ^a^ ± 0.027
WBI	1.036 ^a^ ± 0.006	1.030 ^a^ ± 0.001	1.032 ^a^ ± 0.002	1.029 ^a^ ± 0.011	1.030 ^a^ ± 0.018	1.027 ^a^ ± 0.011	1.031 ^a^ ± 0.013	1.032 ^a^ ± 0.013

**Table 4:** Total chlorophyll content measured with a SPAD chlorophyll meter on the upper and lower sides of *Brassica rapa* subsp. pekinensis growing in different light spectrums and treated with ozone (100 ppb, 21 days) or non-fumigated (control). YL—yellow light (sodium lamp), WL—white light (LED), RGBL—red + green + blue light (LED) and WBL—white + blue light. Average values (±SD) marked with the same letters in the row do not differ significantly according to Duncan’s test, *p* ≤ 0.05, *n* = 7.

Leaf Side	Treatment							
	YL		WL		RGBL		WBL	
	Control	O_3_	Control	O_3_	Control	O_3_	Control	O_3_
Upper	36.63 ^ab^ ± 1.72	35.19 ^b^ ± 1.74	35.56 ^b^ ± 1.77	36.75 ^ab^ ± 1.66	38.50 ^a^ ± 1.73	38.42 ^a^ ± 1.35	35.24 ^b^ ± 1.56	36.28 ^ab^ ± 1.51
Bottom	37.10 ^abc^ ± 1.76	34.22 ^d^ ± 1.67	35.74 ^bcd^ ± 1.65	36.98 ^abc^ ± 1.70	38.15 ^ab^ ± 1.78	38.70 ^a^ ± 1.74	34.66 ^cd^ ± 1.42	36.90 ^abc^ ± 1.68

## Data Availability

Data are contained within the article.

## Supplementary Materials

The following supporting information can be downloaded at https://www.mdpi.com/article/10.3390/ijms23062941/s1. References [64,65,66,67,68] are cited in the Supplementary Materials.
