# Supplementary material for: The Spectral Compositions of Light Changes Physiological Response of Chinese Cabbage to Elevated Ozone Concentration
Source: Int J Mol Sci. 2022 Mar 9;23(6):2941. doi: 10.3390/ijms23062941 (PMC8955156; doi:10.3390/ijms23062941)
Supplement: Supplementary file 1 [file ijms-23-02941-s001.zip › ijms-1624421-SI.pdf]

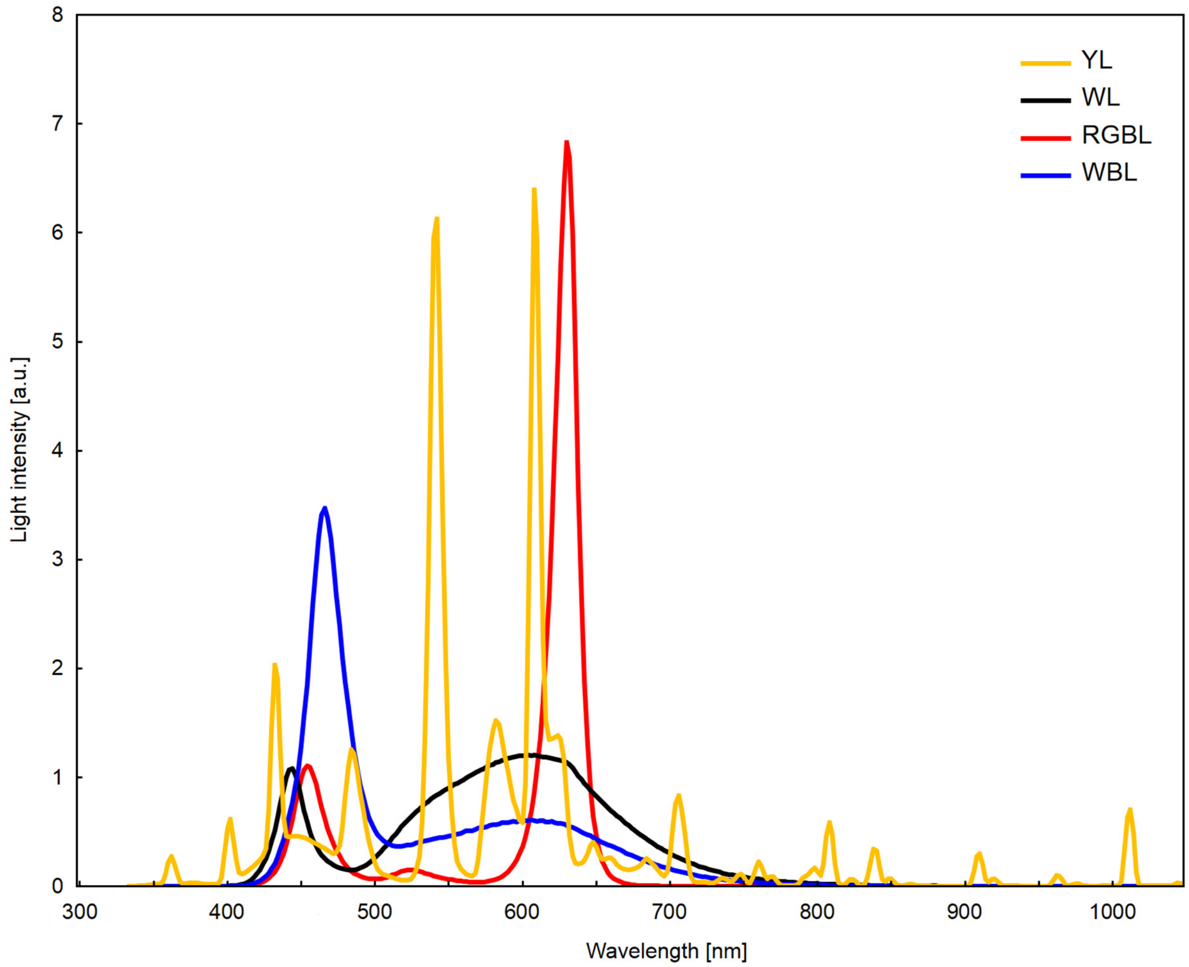

Figure S1. Light spectra used in the experiment: YL—yellow light (sodium lamp), WL—white light (white LED), RGBL—light with the dominant red component (RGB LED), WBL—light with the dominant blue component (white+ blue LED).

Table S1. The characterization of chemical compounds assigned to the particular bands in the FT-Raman spectra obtained from lyophilized leaves of *Brassica rapa* subsp. *pekinensis*

Wavenumber (cm^{-1})	Components	References
746	chlorophyll	Schrader, B., Klump, H. H., Schenzel, K., & Schulz, H. (1999); Vitek, P., Novotna, K., Hodanova, P., Rapantova, B., & Klem, K. (2017).
1005	carotenoids (tetraterpenes)	(Baranska, Schulz, Baranski, Nothnagel, & Christensen, 2005, Vitek et al., 2017, Hartwig Schulz, 2014, Oliwa, Stawoska, Janeczko, Oklestkova, & Skoczowski, 2019;)
1158	carotenoids (tetraterpenes)	(Baranska et al., 2005; Schrader, Klump, Schenzel, & Schulz, 1999, Oliwa, Stawoska, Janeczko, Oklestkova, & Skoczowski, 2019;Hartwig Schulz, 2014; Vitek et al., 2017)
1186	chlorophyll, polyphenols	(Andreev, G., Schrader, B., Schulz, H., Fuchs, R., Popov, S., & Handjieva, N. (2001); Vítek, Novotná, Hodaňová, Rapantová, & Klem, 2017)
1282	chlorophyll;	(Vítek et al., 2017)
1440	lipids, fatty acids, phenolics-phenyl	(Schulz & Baranska, 2007; Vítek et al., 2017)
1525	carotenoids (tetraterpenes)	(Baranski, Baranska, & Schulz, 2005; Schulz & Baranska, 2007; Schulz, Baranska, & Baranski, 2005, Oliwa et al., 2019 Hartwig Schulz, 2014; Vitek et al., 2017; Withnall, Chowdhry, Silver, Edwards, & de Oliveira, 2003;)
1552	Chlorophyll, phenolic compounds	(Heredia-Guerrero et al., 2014; Vitek et al., 2017; Schrader et al., 1999; Schulz & Baranska, 2007)
1602	polyphenols	(Heredia-Guerrero et al., 2014; Schrader et al., 1999; H. Schulz &Baranska, 2007; Vitek et al., 2017)

Andreev, G.; Schrader, B.; Schulz, H.; Fuchs, R.; Popov, S.; Handjieva, N. Non-destructive NIR-FT-Raman analyses in practice. Part 1. Analyses of plants and historic textiles. *Fresenius' Journal of Analytical Chemistry* **2001,** 371(7), 1009-1017.

Baranski, R.,; Baranska, M.; Schulz, H. Changes in carotenoid content and distribution in living plant tissue can be observed and mapped in situ using NIR-FT-Raman spectroscopy. *Planta* **2005**, 222(3), 448-457. doi:10.1007/s00425-005-1566-9

Eravuchira, P. J.; El-Abassy, R. M.; Deshpande, S.; Matei, M. F.; Mishra, S.; Tandon, P.; et al. Raman spectroscopic characterization of different regioisomers of monoacyl and diacyl chlorogenic acid. *Vibrational Spectroscopy* **2012**, 61, 10-16. doi:10.1016/j.vibspec.2012.02.009

Heredia-Guerrero, J. A.; Benitez, J. J.; Dominguez, E.; Bayer, I. S.; Cingolani, R.; Athanassiou, A.; Heredia, A. Infrared and Raman spectroscopic features of plant cuticles: a review. *Frontiers in Plant Science,* **2014**, 5, 14. doi:10.3389/fpls.2014.00305

Oliwa, J.; Stawoska, I.; Janeczko, A.; Oklestkova, J.; Skoczowski, A. Response of the photosynthetic apparatus in the tropical fern Platycerium bifurcatum to increased ozone concentration. *Photosynthetica* **2019**, 57(4), 1119-1129. doi:10.32615/ps.2019.117

Schrader, B.; Klump, H. H.;Schenzel, K.; Schulz, H. Non-destructive NIR FT Raman analysis of plants. *J. Mol. Struct.* **1999**, 509(1-3), 201-212. doi:10.1016/s0022-2860(99)00221-5

Schulz, H. Qualitative and quantitative FT-Raman analysis of plants. In *Optical Spectroscopy and Computational Methods in Biology and Medicine*, M. Baranska (Ed.), Springer: Dordrecht, Netherlands **2014**, pp. 253-278.

Schulz, H.; Baranska, M. Identification and quantification of valuable plant substances by IR and Raman spectroscopy. *Vib Spectrosc.* **2007**, 43, 13-25.

Vitek, P.; Novotna, K.; Hodanova, P.; Rapantova, B.; Klem, K. Detection of herbicide effects on pigment composition and PSII photochemistry in Helianthus annuus by Raman spectroscopy and chlorophyll a fluorescence. *Spectrochimica Acta Part a-Molecular and Biomolecular Spectroscopy* **2017**, 170, 234-241. doi:10.1016/j.saa.2016.07.025

Withnall, R.; Chowdhry, B. Z.; Silver, J., Edwards, H. G. M.; de Oliveira, L. F. C. Raman spectra of carotenoids in natural products. *SpectrochimicaActa Part a-Molecular and Biomolecular Spectroscopy* **2003**, *59*(10), 2207-2212. doi:10.1016/s1386-1425(03)00064-7
